# A quantitative meta-analysis of vitamin C in the pathophysiology of Alzheimer’s disease

**DOI:** 10.3389/fnagi.2022.970263

**Published:** 2022-09-07

**Authors:** Maryam Hamid, Sumaiya Mansoor, Sanila Amber, Saadia Zahid

**Affiliations:** Neurobiology Research Laboratory, Department of Healthcare Biotechnology, Atta-ur-Rahman School of Applied Biosciences, National University of Sciences and Technology, Islamabad, Pakistan

**Keywords:** vitamin C, Alzheimer’s disease, ascorbic acid, amyloid-β, oxidative stress

## Abstract

**Purpose:**

Alzheimer’s disease (AD) is a multifaceted neurodegenerative disorder with many complex pathways feeding into its pathogenesis and progression. Vitamin C, an essential dietary antioxidant, is vital for proper neurological development and maintenance. This meta-analysis and systematic review attempted to define the relationship between vitamin C plasma levels and AD while highlighting the importance and involvement of vitamin C in the pathogenesis of AD.

**Materials and methods:**

PRISMA guidelines were used to obtain studies quantifying the plasma levels of vitamin C in AD and control subjects. The literature was searched in the online databases PubMed, Google Scholar, and Web of Science. A total of 12 studies were included (*n* = 1,100) and analyzed using Comprehensive Meta-Analysis 3.0.

**Results:**

The results show that there is a significant decrease in the plasma vitamin C levels of AD patients as compared to healthy controls (pooled SMD with random-effect model: −1.164, with 95%CI: −1.720 to −0.608, *Z* = −4.102, *p* = 0.00) with significant heterogeneity (*I*^2^ = 93.218). The sensitivity analysis showed directionally similar results. Egger’s regression test (*p* = 0.11) and visual inspection of the funnel plot showed no publication bias.

**Conclusion:**

Based on these studies, it can be deduced that the deficiency of vitamin C is involved in disease progression and supplementation is a plausible preventive and treatment strategy. However, clinical studies are warranted to elucidate its exact mechanistic role in AD pathophysiology and prevention.

## Introduction

Alzheimer’s disease (AD) is an irreversible neurodegenerative disorder associated with memory loss, functional impairment, and a host of psychiatric symptoms such as apathy, agitation, psychosis, and depression (Deardorff and Grossberg, 2019). AD contributes heavily to the global disease burden and is the largest cause of mortality in adults ≥ 65 years of age with over 50°million people living with AD worldwide in 2020 (Zhang et al., 2021). Almost 90% of AD cases are linked to sporadic occurrence, and the remaining cases of AD are inherited. It is a complex multifactorial disease attributed to factors including senescence, amyloid-β (Aβ) plaque formation, oxidative stress, and neuroinflammation (Bekris et al., 2010).

Vitamin C or ascorbic acid (AA; C_6_H_8_O_6_) is a water-soluble organic compound (Padayatty and Levine, 2016). Unlike most mammals, humans and primates lack the essential enzyme L-gulono-γ-lactone oxidase required for the final stage of vitamin C biosynthesis because the mutations in the L-gulono-γ-lactone oxidase (GLO) gene, therefore, are unable to synthesize vitamin C within the body (Lachapelle and Drouin, 2011). Plant sources such as tomatoes, strawberries, citrus fruits, green, and red bell pepper along with other green leafy vegetables are high in vitamin C content (up to 5,000°mg/100°g) (Chambial et al., 2013), while the recommended dietary allowance is 75 mg/day and 90 mg/day for females and males, respectively (Padayatty and Levine, 2016).

Vitamin C has an imperative involvement in the prevention and treatment of a host of diseases and ailments like the common cold, tissue healing, cancer, diabetes, infertility, etc., (Padayatty et al., 2006; Jagetia et al., 2007; Hemilä and Chalker, 2013; Sadeghzadeh et al., 2019; Plevin and Galletly, 2020). In the human brain, the highest concentration of vitamin C is found in the cerebral cortex, hippocampus, and amygdala, while CSF concentrations are higher as compared to plasma (Travica et al., 2017). An increase in the generation of free radicals in various neurodegenerative disorders may indicate that vitamin C could have a prospective role in the therapeutic of such disorders including AD (Kocot et al., 2017). In the current work, we conducted a comprehensive meta-analysis along with systemic review of existing studies examining vitamin C in AD.

### Vitamin C and brain

The intense physiochemical activity of the brain requires a high metabolic rate and consumption of copious amounts of oxygen and glucose. The high metabolic rate and enzymatic processes lead to the generation and accumulation of free radicals, most importantly reactive oxygen species (ROS) (Watts et al., 2018; Jelinek et al., 2021). Maintaining the delicate oxidative balance depends on antioxidants in the brain of which vitamin C has the highest concentration (Kaźmierczak-Barañska et al., 2020). The strong reducing property of AA can be attributed to the hydroxyl groups present in the lactone ring that serve to either donate electrons or protons. The hydroxyl groups react with hydroxyl radicals, peroxide radicals, hydrogen peroxide, etc.; as a result, AA is oxidized to dehydroascorbate, a bicyclic hemiketal which may be reduced back to AA by the enzyme dehydroascorbate reductase (Englard and Seifter, 1986; Njus et al., 2020).

Vitamin C is essential for neurodevelopment, neurotransmitter regulation, and glutamate-mediated neurotransmission and to maintain oxidative balance. It is a vital nutrient for brain function particularly due to its antioxidant properties. It regulates the biosynthesis of catecholamine’s dopamine and norepinephrine and mediates the dopamine neuron differentiation through TET1- and JMJD3-dependent mechanism in the fetal midbrain indicating an epigenetic role (He et al., 2015). It is subsequently involved in the conversion of dopamine to norepinephrine by acting as an electron donor for dopamine β-hydroxylase (DβH) and prevents dopamine-mediated superoxide formation (May et al., 2013). Vitamin C also promotes DNA demethylation of pro-myelinating genes that result in the regulation of Schwann cell myelination (Huff et al., 2021).

The major excitatory neurotransmitter responsible for relaying messages in the CNS is glutamate. It binds to metabotropic NMDA receptors, activating them, and inducing calcium influx into neuronal cells. Dysregulation of glutamate concentration leads to a disturbance in neuronal calcium homeostasis that leads to neuronal damage and cell death in several neurological disorders (Lewerenz and Maher, 2015; Shah et al., 2015). Persistent elevation in intracellular calcium levels induces apoptosis by either activating apoptotic enzymes such as calpain, inhibiting normal cellular protein synthesis due to depletion of endoplasmic reticulum calcium levels, or by destroying the mitochondrial membrane potential (Demaurex and Distelhorst, 2003). Vitamin C is effective against glutamate-mediated cytotoxicity by reversible inhibition of NMDA activity and reduces the glutamate-induced phosphorylation of AMPK (Majewska et al., 1990; Shah et al., 2015). It is evident that vitamin C ameliorates the glutamate-induced neurotoxicity *via* the ascorbate–glutamate heteroexchange that keeps the excitatory overload of glutamate in check. Glutamate uptake results in the release of vitamin C in the extracellular fluid that later removes the glutamate-generated ROS. However, deficiency of ascorbate dysregulates glutamate clearance leading to its accumulation and ultimately excitotoxicity of neurons that contributes to cognitive impairment (Mi et al., 2018).

### Vitamin C and Alzheimer’s disease

The formation and deposition of neurofibrillary tangles and Aβ plaques are characteristics of an AD patient’s brain. These depositions are highly insoluble and are composed of densely packed filaments (Bloom, 2014). The proteolytic cleavage of amyloid precursor protein (APP) directs the formation of Aβ plaques, while the hyperphosphorylation of tau protein tends to generate neurofibrillary tangles. These cellular events can be affiliated with oxidative stress, although it is still ambiguous whether oxidative stress acts as an early or late event in the pathogenesis of AD (Apelt et al., 2004). The facilitation of amyloidogenic pathway of APP processing by oxidative stress leads to enhanced production and deposition of Aβ. Followed by oxidative stress, the increased activity of β-secretase is conceivably facilitated through phosphorylation of p42/44 MAPK (Muche et al., 2017).

**FIGURE 1:**
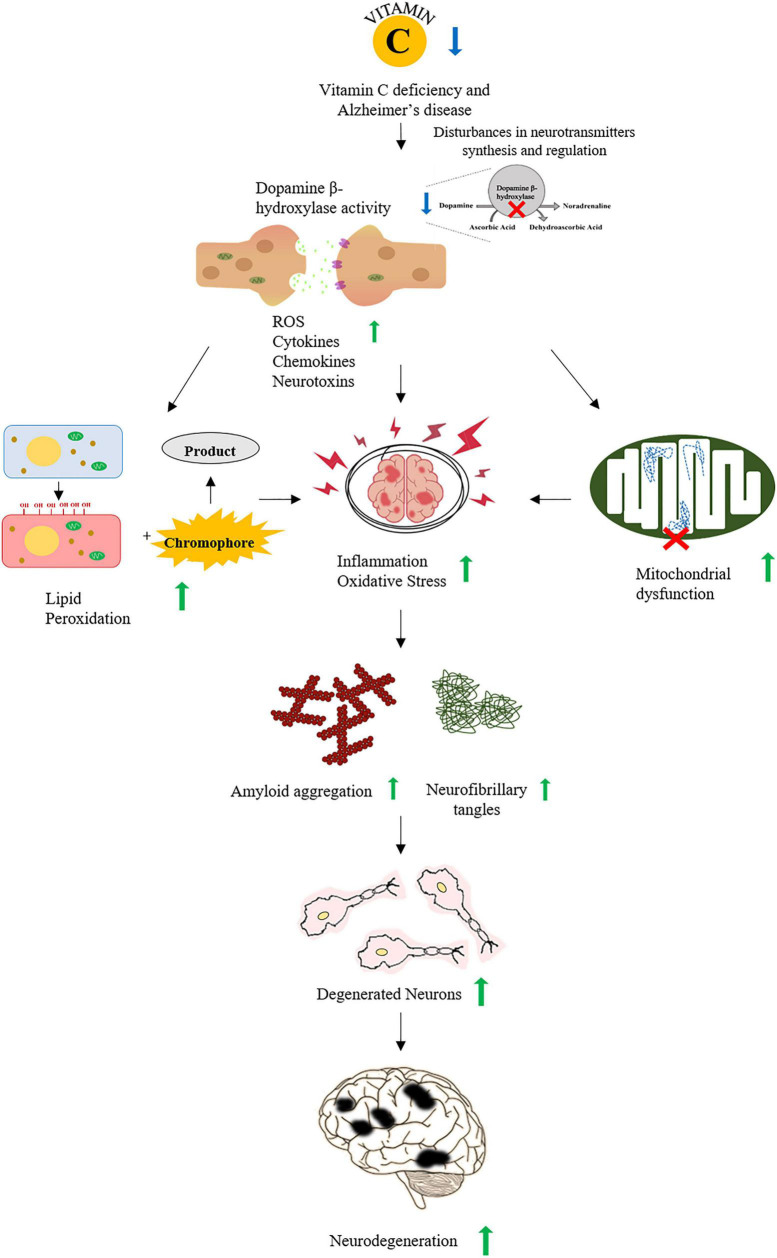
A schematic representation of association between vitamin C and Alzheimer’s disease (AD). Vitamin C deficiency interrupts the synthesis and regulation of neurotransmitters increasing ROS, cytokines, and neurotoxins that lead to increased oxidative stress and inflammation. Oxidative stress also increases the accumulation of Aβ plaques and the formation of neurofibrillary tangles. All of these events promote the neurodegeneration observed in AD.

Ascorbic acid (AA) plays a major role in AD pathophysiology due to its antioxidant and neuroprotective property particularly against ROS (Covarrubias-Pinto et al., 2015; Figure 1). It is released by the glial cells into the synaptic clefts of neurons in CNS (Röhl et al., 2010) and is responsible for encouraging the regeneration of antioxidant enzymes including glutathione (GSH) and catalase (Dringen et al., 1999). Several studies showed the potential use of AA as a therapeutic agent to stall the progression of AD. A study in APP/PSEN 1 transgenic mice served as evidence for nootropic abilities of AA. Mice injected parenterally with AA showed enhanced cognitive abilities despite no altering of oxidative stress and plaque deposition (Harrison et al., 2009). Another study suggested that the oral intake of AA reduced the Aβ fibrils-mediated oxidative stress (Rosales-Corral et al., 2003). Since the Aβ plaques contain metal-binding sites for zinc, iron, and copper (Bush et al., 2003), these bound metals can stabilize the Aβ plaques and increase the rate of deposition and cytotoxicity (Jomova et al., 2010). The metal redox activity of active iron and copper leads to the production of peroxynitrites, hydroxyl ions, carbonyls, and advanced glycation end products (AGEs). The hydroxyl ions generated by metal redox reactions increase the lipid peroxidation, DNA, and protein oxidation. The production and interaction of AGE with its cell membrane-bound receptors further lead to proinflammatory responses (Yao et al., 2004). However, the soluble forms of receptor for AGEs (RAGE) inhibit the binding of ligands to the cell membrane-bound RAGE, thus inhibiting the neurotoxic or proinflammatory responses (Lue et al., 2009).

The post-mitotic nature of neurons and their higher levels of oxygen requirement make the brain highly vulnerable to oxidative stress (Mecocci et al., 2002). Likewise, oxidative stress may activate microglia and astrocytes at the site of higher oxidative stress (García-Krauss et al., 2016) where the interaction of glial cells with neurons produces inflammatory cytokines, nitric oxide, and chemokines which drives the inflammation in the nervous system (Sultana et al., 2011). A few studies have emphasized the role of AA as a pro-oxidant due to the interaction of toxic forms of Aβ with AA resulting in the formation of −OH radicals (Valko et al., 2005). However, a recent finding demonstrates ascorbic acid-mediated destabilization of preformed amyloid fibrils and protection against amyloid-induced cytotoxicity (Alam et al., 2017).

Oxidative stress as a major epigenetic factor induces mitochondrial dysfunction that extensively contributes in AD as alterations in the energy metabolic pathways are witnessed (Perez Ortiz and Swerdlow, 2019). Salient observations include glucose hypometabolism due to reduced glycolysis and compensation of energy deficit by using fats and amino acids as an alternate energy source (Toledo et al., 2017). Several glycolytic and mitochondrial proteins exhibit altered levels due to oxidative modifications that perturbed the glucose metabolism in AD. These modified proteins also contribute toward dysregulation of various other biochemical and metabolic pathways altered in AD (Butterfield and Boyd-Kimball, 2018).

Similarly, ROS-mediated mitochondrial dysfunction may further damage mitochondrial membrane permeability and respiratory chain and induce mitochondrial DNA mutations (Guo et al., 2013). AA prevents mitochondrial membrane depolarization and ultimately oxidative injury by accessing the mitochondria in its oxidized form through glucose transporter 1 (Glut1) (Kc et al., 2005). Nonetheless, the complex mechanism and regulation of AA uptake by the organelles are attributed to its diversified antioxidant property that may alleviate the mitochondrial dysfunction by quenching the mitochondrial ROS (Fiorani et al., 2021). Moreover, various genes associated with cellular mechanisms of oxidative damage repair may serve as important candidates for AD. For instance, silent information regulator type-1 (SIRT1) which is involved in various cellular processes including cell survival, control of apoptosis, and modulation of ROS levels showed a positive association with the AD and is linked to pathways that may impair oxidative stress (Camporez et al., 2021). The upregulation of SIRT1 antagonizes AD through improvement in the oxidative balance (Xu et al., 2020). Similarly, the regulation of uncoupling protein 2 (UCP2) by stanniocalcin-1 (STC-1) alleviates oxidative stress and Aβ level as observed in the N2a/APP695s we cells treated with oleanolic acid (Guo et al., 2020). In addition, recent evidence on the involvement of NF-E2-related factor 2 (Nrf2), a stress-responsive transcription factor, ameliorates cognitive impairment by suppressing oxidative stress, and neuroinflammation observed in *App* knock-in AD mouse model (Uruno et al., 2020). Likewise, microRNA-23b attenuates tau pathology and inhibits oxidative stress by targeting N-acetylglucosaminyltransferase II (GnT-II) in AD (Pan et al., 2021). It is also noteworthy that NADPH oxidase 4 (NOX4) mediates the ferroptosis of astrocytes by oxidative stress-induced lipid peroxidation through the impairment of mitochondrial metabolism in AD (Park et al., 2021). Collectively, these modulating factors are crucial candidates for AD and highlight a strong association of oxidative stress with AD pathology.

Vitamin C is vital for antioxidative mechanism and is extremely important for homeostasis and the proper functioning of the CNS. Besides general free radical trapping, the suppression of proinflammatory genes, neuroinflammation, and Aβ fibrillogenesis is also emerging roles of vitamin C (Monacelli et al., 2017; Kaźmierczak-Barañska et al., 2020). Although the concentration of vitamin C as ascorbate is higher in the CNS, however, the plasma levels are indicative of various key processes associated with CNS. For instance, the age-associated differences in plasma and brain vitamin C are linked to age-associated cognitive differences, alongside compromised vitamin C brain regulation (Travica et al., 2020). Interestingly, the high plasma vitamin C status also elevates overall mood in young adult males (Pullar et al., 2018). Taking into consideration the vital role of vitamin C, this meta-analysis was conducted to establish whether there was a statistically significant difference in the plasma levels of vitamin C/AA in AD patients and healthy controls. This information can be useful to determine if vitamin C supplementation interventions are a valuable avenue to look into as it may corroborate the role of AA in AD pathology.

## Materials and methods

### Search strategies and selection of studies

The purpose of this study was to describe the importance of vitamin C with relevance to the neurological disorder AD and elucidate the association of AD with vitamin C levels. The studies included were obtained according to Preferred Reporting Items for Systematic Reviews and Meta-Analyses (PRISMA) guidelines (Moher et al., 2009).

The literature was searched in the online databases PubMed, Google Scholar, and Web of Science using the keywords “Alzheimer’s disease (AD),” “vitamin C,” and “ascorbic acid (AA).” The timeline of the searched literature was set for the last 21 years ranging from 2000 to January 2022.

### Inclusion criteria

Those studies were identified that contained “Alzheimer’s disease” (AD), “vitamin C,” and “ascorbic acid” (AA) in the title, abstract, or key descriptors. The selection criteria included: human subjects, non-randomized observational studies (case-control and cross-sectional), neuropsychological testing of subjects to determine AD, mean or median plasma levels of vitamin C as one of the main variables of interest, and the study should compare two groups of interest (AD patients vs. controls). Certain articles contained information on several antioxidants and micronutrients; in such cases, only the information about mean vitamin C levels was retrieved. The English language was used as a restriction during data collection.

### Exclusion criteria

Studies were excluded if they were unrelated to plasma vitamin C levels or AD and if they were animal studies or reviews. Studies that reported plasma vitamin C levels without comparison to a healthy control group were excluded. Studies that did not report the median or mean values were also excluded. For those studies which used the same cohort to publish several articles, only the article having the maximum number of participants was included. In the end, six studies were included according to the inclusion and exclusion criteria.

### Statistical analysis

Comprehensive Meta-Analysis Version 3.0 was used to perform the statistical analysis (Borenstein et al., 2013). To account for heterogeneity, the random-effects model was used. Standardized mean differences (SMDs) were calculated for differences in means between AD patients and healthy controls. Forest plots were generated, and 95%CI (confidence interval) was also included. The *I*^2^ value was used as a measure of heterogeneity. For *I*^2^ ≥ 75%, which suggests high heterogeneity, meta-regression was performed to identify the sources contributing to heterogeneity. Publication bias was determined using Egger’s regression tests (Egger et al., 1997). Sensitivity analysis was also conducted using the one-study removal method in the Comprehensive Meta-Analysis Version 3.0. The Cochrane Collaboration’s tool for Risk Of Bias in Non-randomized Studies–of Exposure (ROBINS-E) was used for each observational study included in the analysis (Bero et al., 2018; Robins-E Development Group, Higgins et al., 2022). The evaluation includes confounding factors, selection of participants, misclassification of variables, bias due to missing data, and reverse causation. These were evaluated as either high or low risk of bias; if the studies were unclear regarding the data, risk of bias was indicated as unclear.

## Results

### Selection of studies

**FIGURE 2:**
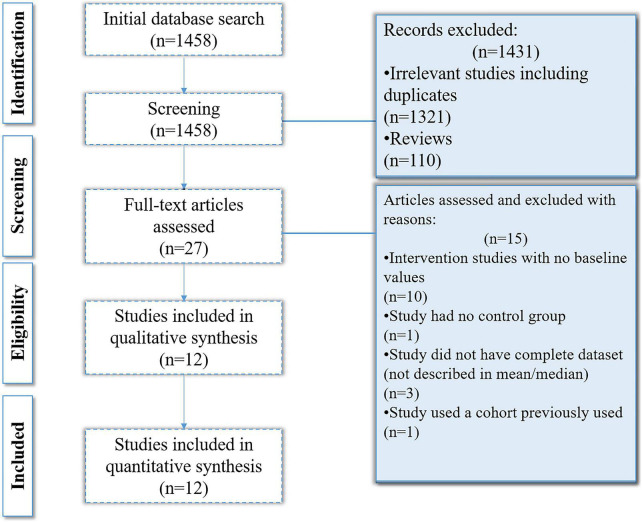
Flowchart of the selection process.

The online databases search yielded 1,458 results out of which 1,331 were removed on the basis of irrelevancy and not meeting inclusion criteria. On the basis of title and abstract relevancy, 27 studies were selected which were further scrutinized. Fifteen of the 27 studies were removed as they either had no control groups, were intervention studies that lacked baseline values, did not have a complete data set, or the cohort was used in a previously published article. Ultimately, 12 studies were selected and included in the analysis (Figure 2).

Data collection was performed in a hierarchical manner. The articles were downloaded, reviewed, and filtered based on the inclusion and exclusion criteria. All such studies that were reporting the deficiency of plasma vitamin C levels in AD are summarized in Table 1.

**TABLE 1:** Alzheimer’s disease (AD) to control ratio for vitamin C.

			Number of		Mean age		Vit C conc. in AD	Vit C conc. in	
Studies	Type	Disease	participants		(years)		(μmol/L)	cont. (μmol/L)	Implications
							
			AD	Cont.	AD	Cont.			
Schippling et al. (2000)	Case-control study	AD	29	29	71.7 ± 10.1	55.1 ± 18.8	35.0 ± 18.6	48.8 ± 18.5	An increase in lipoprotein oxidizability and lower levels of AA in AD implicate oxidation in the pathogenesis of AD.
McGrath et al. (2001)	Case-control study	AD	29	46	74 ± 7.75	73 ± 5.75	9.9 ± 6.9	24.2 ± 8.6	The serum antioxidant levels of AD patients were considerably lower as compared to the control group. (*p* < 0.05)
Polidori and Mecocci (2002)	Case-control study	AD	35	40	85.9 ± 5.5	85.5 ± 4.4	18.1 ± 5.8	35.9 ± 6.3	AD patients exhibit statistically significant (*p* < 0.001) poorer levels of AA when compared to controls.
Mecocci et al. (2002)	Case-control study	AD	40	39	75.9 ± 5.4	74.8 ± 6.3	32.28 ± 10.8	56.74 ± 15.9	Biomarkers of oxidative damage are increased, and antioxidant levels are decreased in AD.
Rinaldi et al. (2003)	Case-control study	AD	63	56	76.8 ± 6.9	75.8 ± 7.2	25.9 ± 8.9	52.4 ± 16.5	Plasma levels and activity of AA were depleted in AD patients.
Quinn et al. (2003)	Case-control study	AD	10	10	65 ± 7	66 ± 6	58.1 ± 42	86.4 ± 39	The trend of lowered plasma vitamin C in AD patients was observed.
Glasø et al. (2004)	Case-control study	AD	20	18	75–85	75–85	46.2 ± 25	77.7 ± 28	Significant difference was observed between the AA levels of AD and control subjects.
Polidori et al. (2004)	Cross-sectional study	AD	63	55	76.8 ± 6.9	75.7 ± 7.3	25.9 ± 8.9	52.4 ± 16.4	Regardless of the nature of dementia, i.e., vascular or neurodegenerative, it is associated with a drastic decrease in the blood antioxidant level.
Giavarotti et al. (2013)	Case-control study	AD	23	42	82	82	52 ± 23.9	48 ± 19.4	Antioxidant levels are disturbed in AD along with an activation of the inflammatory pathways.
Mangialasche et al. (2015)	Cross-sectional study	AD	28	21	74.9 ± 6.9	79.1 ± 7.7	23.6 ± 3.5	25.9 ± 2.8	Mitochondrial dysfunction is observed in AD patients, and this dysfunction is correlated with plasma antioxidant levels.
Ulstein and Bøhmer (2017)	Cross-sectional study	AD	48	63	71 ± 8.2	72.7 ± 6.3	62.8 ± 28.9	62.8 ± 17.8	No significant difference was observed between the groups.
Lanyau-Domínguez et al. (2021)	Cross-sectional study	AD	43	250	78	82.8	61.6 ± 51.9	79.1 ± 64	Vitamin deficiency was observed in AD compared to controls.

### Meta-analysis of vitamin C concentrations in plasma

**FIGURE 3:**
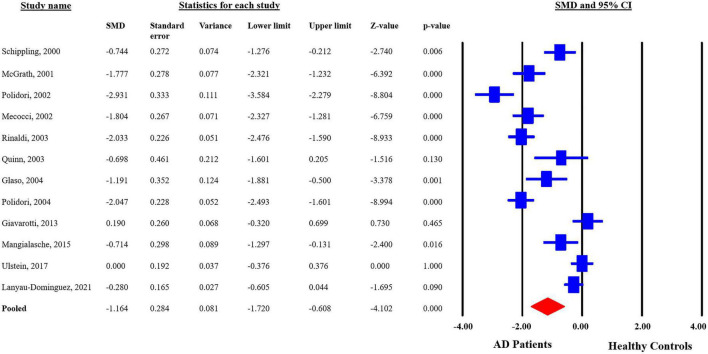
Forest plot for plasma concentrations of vitamin C in Alzheimer’s disease (AD) patients and healthy controls. CI, confidence interval; SMD, standardized mean difference.

The analyzed data from the selected studies showed that plasma vitamin C levels are significantly reduced in the AD patients as compared to the healthy controls (pooled SMD with random-effect model: −1.164, with 95%CI: −1.720 to −0.0608, *Z* = −4.102, *p* = 0.00). The heterogeneity in-between studies were significant (*Q* = 162.191, d*f* = 11, *p* = 0.00, *I*^2^ = 93.218). The *p*-value indicates that the dispersion is not due to random error, but in fact, it is due to real differences in the study effects (Figure 3). Sensitivity analysis showed that no single study greatly impacted the results of the analysis and there were no outliers.

### Publication bias

**FIGURE 4:**
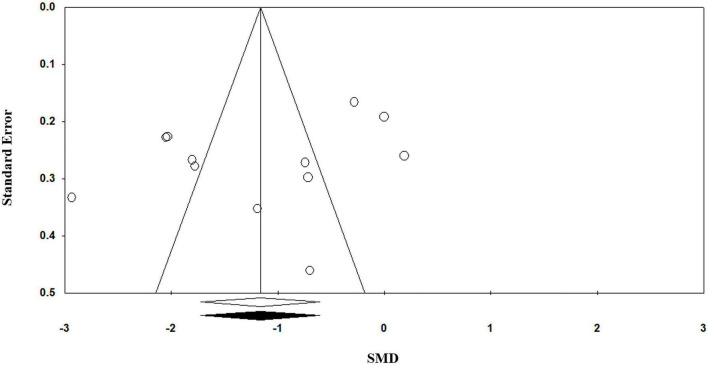
Funnel plot for the meta-analysis regarding the association between vitamin C deficiency and Alzheimer’s disease (AD).

Egger’s regression test (*p* = 0.11) suggests no publication bias in the meta-analysis since *p* > 0.05 indicates no publication bias. Visual inspection of the funnel plot symmetry also corroborates this result (Figure 4).

### Risk of bias

**FIGURE 5:**
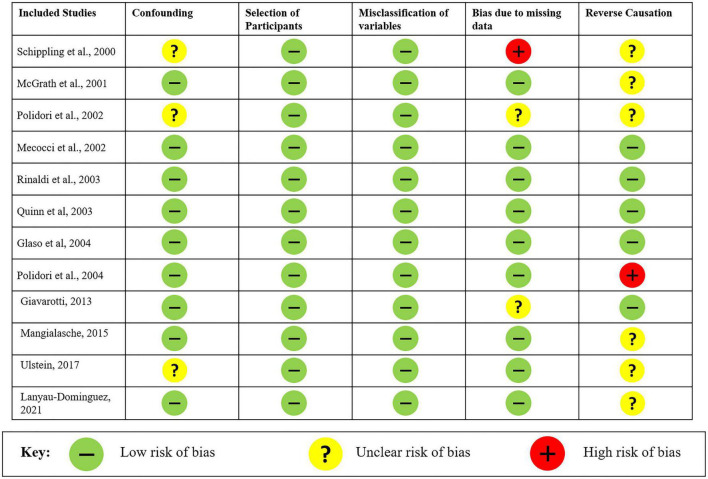
Risk of bias analysis of studies evaluated using Cochrane’s Risk Of Bias tool in Non-randomized Studies–of Exposures (ROBINS-E).

The risk of bias and the quality of the observational non-randomized studies were analyzed in the meta-analysis through Cochrane’s Risk Of Bias tool in Non-randomized Studies–of Exposure (ROBINS-E). Overall, the risk of bias in the analyzed studies is “low risk of bias except for concerns regarding residual confounding.” The studies reported all possible variables that could influence the levels of vitamin C along with a single study having missing data for comorbidities of the patients. Most of the studies were unclear regarding the causation and concluded as decrease in plasma vitamin C levels was accompanied by or leading to AD along with a single study reporting a decrease in the antioxidant levels. The risk of bias for all 12 studies is shown in Figure 5.

## Discussion

This study was designed to compare the plasma and serum levels of vitamin C in AD patients with healthy controls. Results of this meta-analysis show that the levels of vitamin C in AD patients were significantly decreased (12 studies, *n* = 1,100). A significant heterogeneity was also evident. To account for heterogeneity, three major moderators were identified which included latitude, age of patients, and percentage of females within the study. Latitude is an important moderator as dietary habits change according to the location of the test subjects and may have an effect on the study results. The age and sex of subjects were also taken into account. Due to missing data, comorbidities, and MMSE could not be applied, however, might be involved in the heterogeneity shown in the study. The *R*^2^ value is 0.31 which indicates that 31% of the heterogeneity is accounted for when all three of the moderators are checked for heterogeneity.

Although when the moderators were individually checked for heterogeneity, latitude, and sex accounted for most of the heterogeneity in the model with *R*^2^ values 0.10 and 0.22, respectively, indicating that 10 and 22% of the heterogeneity in the model being the result of variation in the latitude and sex of the patients. The heterogeneity shown by latitude is 10% which can be attributed to the fact that the conducted studies spanned a wide region; therefore, a considerable variation in the diet is anticipated (Podcasy and Epperson, 2022).

Age did not explain the heterogeneity in the model represented by a zero *R*^2^ value (0.00) indicating the presence of 0% of the heterogeneity in the model. Age is not involved in the causation of AD as depicted by the zero contribution of age to heterogeneity in the analysis; however, previous studies have shown that the risk of AD increases with age (Guerreiro and Bras, 2015). One of the studies included patients with major comorbidities such as coronary heart disease, diabetes, and hypertension, while all other studies excluded patients that had any comorbidity or smoked. In addition, variation in MMSE scores of patients could be another potential factor as each study used a different range or threshold to include patients in the trial leading to diversity in the level of cognitive impairment (Mitchell, 2009; Nagaratnam et al., 2020).

There is no publication bias present in the meta-analysis. Most of the studies have taken the potential confounders such as age, gender, diet, MMSE, comorbidities, etc., under consideration with two studies that did not account for comorbidities. The sensitivity analysis was conducted to see whether an outlier significantly affected the results, and the analysis showed that there was no single study that greatly impacted the results of the meta-analysis. The combined SMD remained consistent even after the removal of studies separately.

The earliest study included in this review, published in 2000, assessed the cerebrospinal fluid (CSF) and plasma vitamin C level in AD patients versus healthy controls and the lipid oxidizability in AD patients, to establish a link between lipid oxidation, plasma antioxidants, and AD progression. The vitamin C levels were significantly decreased in CSF and plasma of AD patients. This implies that the increased lipid oxidizability could be involved in the pathogenesis of AD (Schippling et al., 2000). Vitamin C plasma levels are considered critical for the onset and the progression of aging and AD (Covarrubias-Pinto et al., 2015). Therefore, various studies tried to establish the link between peripheral levels of vitamin C and disease progression as well as the result of antioxidant supplementation.

Oxidative stress has been strongly associated with the process of neurodegeneration and cognitive decline. Due to the increasing concentration of ROS and the decreasing neutralization activity of the antioxidants, oxidative stress causes irreversible neuronal damage and apoptosis (Guo et al., 2013). The crucial players involved in neutralizing ROS are enzymatic (SOD and GPx) and non-enzymatic antioxidants (vitamin E and vitamin C). Vitamin C is a free radical scavenger and donates two of its electrons that prevents the oxidation of other more harmful substances. A small quantity of the resulting compound dehydroascorbate is converted back to AA *via* reduction, and the rest is metabolized to oxalate by hydrolysis (Covarrubias-Pinto et al., 2015).

A case-control study assessed the increased oxidative stress in AD through quantitation of peripheral marker, a lipid peroxidation product 4-hydroxynonenal (4-HNE), and the plasma levels of AA and vitamin E along with sulfhydryls. There was a significant increase in levels of 4-HNE, while AA was substantially decreased in AD patients in comparison to healthy controls (McGrath et al., 2001). 4-HNE is considered to elevate γ-secretase activity, induce Aβ aggregation, and promote protofibril formation. The marked reduction in AA levels may indicate a possible failure to detoxify 4-HNE, exacerbating the oxidative stress and progression of AD (Siegel et al., 2007; Zhang et al., 2018).

Similarly, assessment of the levels of a wide range of antioxidants including vitamin C and the extent of lipid peroxidation revealed decreased plasma antioxidant levels in AD patients, while the difference in the level of vitamin C was also statistically significant indicating the susceptibility of AD patients to oxidative insult (Polidori and Mecocci, 2002). A case-control study revealed an increased consumption of antioxidants in the brain *via* the evaluation of CSF to plasma ratio of vitamin C (Quinn et al., 2003). Another study that evaluated the nutritional factors associated with late-onset dementia of the Alzheimer’s type concluded that the decreased levels of several vitamins including vitamin C may contribute to the development of AD (Glasø et al., 2004).

Moreover, the biomarkers of oxidative damage to DNA such as 8-hydroxy-2’-deoxyguanosine on lymphocytes are also increased in AD along with a decline in plasma antioxidants and vitamin C, A, and E as well as carotenoids such as lutein, α-carotene, β-carotene, and lycopene (Mecocci et al., 2002). This further depicts that increased oxidative stress is related to a poor antioxidant status in AD (Fracassi et al., 2021). A decrease in the overall activity of enzymatic antioxidants along with reduced plasma levels of non-enzymatic antioxidants including vitamin C in AD and MCI patients was also reported. The study attributed the poor plasma concentrations of antioxidants as a result of rapid depletion through neutralizing free radicals produced due to oxidative stress (Rinaldi et al., 2003). A cross-sectional study evaluated the plasma antioxidant levels in patients with AD and vascular dementia. The study concluded that irrespective of the nature of dementia whether the cause is vascular or neurodegenerative in nature, both suffer from a drastic decrease in the blood antioxidant levels when compared with the controls (Polidori et al., 2004).

As AD is accompanied by increased inflammation and oxidative stress, another case-control study explicated a higher activation of circulation monocytes and inflammatory markers in AD along with a decrease in the circulating vitamin C and α-tocopherol levels (Giavarotti et al., 2013). Similarly, reduced mitochondrial aconitase activity is observed in AD patients which leads to mitochondrial dysfunction which was correlated with the decrease of plasma antioxidants (Mangialasche et al., 2015). Likewise, a cross-sectional study by Ulstein and Bøhmer (2017), explored the association between deficiencies of several vitamins and AD. Although their results do not support the notion that vitamin deficiencies are not involved in the causation of AD, perhaps it can be due to the limitations of the study as they did not account for whether the participants were taking vitamin supplements or not and the patients’ age was comparatively younger than the general AD population (Ulstein and Bøhmer, 2017). Contrarily a recent cross-sectional study on Cuban older adults concluded that hyperhomocysteinemia and vitamin deficiencies are associated with AD (Lanyau-Domínguez et al., 2021).

Although the 12 studies explored relative quantities of different antioxidants and oxidative stress markers in plasma of AD patients when compared to healthy controls, a consistent decrease in the plasma vitamin C concentration of AD patients was observed across all studies. This marked reduction in plasma vitamin C levels may be attributed as a possible contributing factor for the causation of AD since the substantial decrease in the antioxidant level would lead to increased oxidative stress, neuroinflammation, Aβ fibrillogenesis, and aberrations in various other molecular processes regulated by vitamin C.

### Intervention studies

As elevated levels of ROS are a characteristic of AD and accelerate disease progression, dietary antioxidants are vital to offset the detrimental effects of oxidative stress. Dietary antioxidants such as vitamin C can decrease ROS level thereby decelerating disease progression. Although the dosage is not optimized, a diet rich in vitamin C may benefit AD patients as evident through various observational studies and clinical trials (Polidori and Nelles, 2014).

A cohort study with 5,395 participants was examined for incident dementia over a period of 6°years. The data revealed that 197 of the participants developed dementia out of which 146 had AD. The study observed that even after adjusting for factors such as age, sex, body mass index, base pack years of smoking, etc., the high intake of vitamin E and C decreased the risk of AD (Engelhart et al., 2002). Another cross-sectional study showed that vitamin C, E, or a combination of both decreased the prevalence of AD (Zandi et al., 2004).

Although the mechanism through which vitamin C delays AD progression is not properly described, vitamin C supplementation is involved in the proteolytic processing of APP and leads to a decrease in the Aβ peptides along with a substantial reduction of lysosomal enzymes in sporadic AD as shown by the effects of supplementing 50°uM vitamin C to skin fibroblasts of AD patients (Costanzi et al., 2008). Similar results were obtained through animal model studies where increased supplementation of vitamin C of 3.3°g/l through drinking water reduced the Aβ plaque burden in the hippocampus and cortex of KO-Tg mice, alleviating the mitochondrial aberrations and BBB disruption (Kook et al., 2014). Whereas, APP + PSEN1 transgenic mice when fed a blueberry supplement diet for 8°months showed no memory and learning deficits although there was no change in the Aβ deposition (Joseph et al., 2003). Interestingly, even a mild deficiency of vitamin C may accelerate amyloid pathogenesis *via* modulation of oxidative stress mechanism contributing toward impaired cognition (Dixit et al., 2015). Treatment with solution of vitamin C for a period of 6°months demonstrated a significant decline in ROS, Aβ peptides, and synaptophysin in an AD mouse model (Murakami et al., 2011).

Evaluation of nutrient intake of elderly community-dwelling AD patients showed poor dietary intakes associated with significant differences in levels of macronutrients, energy, zinc, calcium, iron, etc., (Shatenstein et al., 2007). Another similar report including 8,085 participants over a 4-year period showed that consumption of fruit and vegetables decreased the risk of AD and dementia (Barberger-Gateau et al., 2007), while increased intake of vitamin C-rich food like strawberries and star fruit substantially curtails the risk of developing AD by minimizing the oxidative stress and inflammation (Leelarungrayub et al., 2016a,b; Agarwal et al., 2019). A cohort study evaluated the effect of antioxidants when combined with a cholinesterase inhibitor, i.e., donepezil in AD. The group which was supplemented with antioxidants showed a significant reduction in homocysteine levels and number of sickle erythrocytes as compared to placebo. Increase in GSH levels was strongly correlated with improvement in MMSE II scores. The improvement corresponded to a decrease in oxidative stress in antioxidant combined with donepezil-treated group (Cornelli, 2010). Goschorska et al. (2018) observed a significant suppression in the antioxidant action of AChE inhibitors; however, it is postulated that supplementation of antioxidants like vitamin C may improve the clinical consequences associated with oxidative stress in AD. Lipids and lipoproteins are the main targets of oxidation in the brain. Combined supplementation of vitamin E and C decreased the susceptibility of lipoproteins for oxidation in AD patients, essentially inhibiting lipid peroxidation in the brain (Kontush et al., 2001; Arlt et al., 2002). The key observations of these interventional studies are presented in Table 2.

**TABLE 2:** Interventional studies on the status of vitamin C and Alzheimer’s disease (AD).

Study	Type	Participants	Vitamin C intake (diet or supplements)	Follow-up/Treatment period	Effects	Result
Kontush et al. (2001)	Cohort study	20 AD patients were divided into two groups	Vitamin C supplementation 1,000°mg/d	1°month	Effect on antioxidants and lipoproteins.	Supplementation increases plasma and CSF antioxidants while decreasing plasma lipoproteins.
Engelhart et al. (2002)	Cohort study	5,395 participants	Vitamin C average measurement 121.6 mg/d (baseline)	6 years	197 participants developed dementia and 146 developed AD.	High intake of vitamin C through diet was associated with low incidence of AD.
Zandi et al. (2004)	Cross-sectional study	4,740 participants with 200 AD patients	Supplemental use of vitamin C 500°mg/d	2 years	104 more participants developed AD.	Vitamin C intake was lower in participants that developed AD.
Barberger-Gateau et al. (2007)	Cohort study	8,085 participants	Fruit consumption	4 years	183 developed AD and 281 developed dementia.	Frequent consumption of fruit and vegetables decreases the incidence of AD.
Cornelli (2010)	Cohort study	52 AD patients were divided into two groups	Multivitamin supplementation plus donepezil	6 months	Effect on oxidative stress.	Significant decrease in oxidative stress and homocysteine levels was observed.
Galasko et al. (2012)	Randomized controlled trial	78 AD subjects were divided into three groups	Vitamin C supplementation 500°mg/d	16 weeks of treatment period	Effect on oxidative stress.	Supplementation decreased oxidative stress in the brain of AD patients.
Leelarungrayub et al. (2016a)	Preliminary study	29 elderly participants	Starfruit consumption 100°g daily	4 weeks	Effect on inflammatory cytokines.	Consumption decreases proinflammatory cytokines.
Leelarungrayub et al. (2016b)	Preliminary study	27 elderly participants	Starfruit consumption 100°g daily	2 weeks	Effect on blood antioxidants.	Consumption increased antioxidant levels.
Agarwal et al. (2019)	Cohort study	925 participants	Strawberry consumption	6.7 years	245 participants developed AD.	Consumption decreased the incidence of AD.

A randomized clinical trial was conducted between 2006 and 2008 that involved 78 participants with diagnosis of AD divided into three groups with 26 participants each. The treatment group was supplemented with a combination of vitamin C, vitamin E, and alpha-lipoic acid in capsule form for 16 weeks, while the other two were given coenzyme Q and placebo, respectively. The results revealed that although the vitamin C group did not show any change in CSF Aβ42 or tau levels, oxidative stress was substantially decreased and remained unchanged in the other groups, comparatively. Further, longer trials would be required to corroborate their results (Galasko et al., 2012). Another ongoing clinical trial evaluating the effect of lifestyle changes on AD patients recruited 100 AD patients that were randomly divided into two groups. The interventional group received changes in diet including vitamin C supplementation. Their primary purpose is to reduce or possibly stop the progression of AD *via* such changes (Cornelli, 2010; Ornish, 2020).

### Limitations and future directions

Vitamin C as an essential antioxidant mitigates the damage caused by ROS. The reduced concentration of vitamin C in the plasma of AD patients reiterates the involvement of ROS in the incidence and progression of the disease. Treatment protocols involving supplementation and dietary enrichment of vitamin C reduced disease progression and alleviated symptoms of AD patients. The supplementation studies also demonstrated reduction in risk of disease in subjects consuming high doses of vitamin C supplements or high consumption of fruits and vegetables. Although one of the limitations of this meta-analysis is that the studies included were conducted in neighboring European countries with similar latitudes, it is recommended to conduct studies from other regions so as to diversify the findings. Diversification of studies will help to find the link between diet, genotype, and disease link.

## Conclusion

In conclusion, this meta-analysis suggests that the consumption of vitamin C may be used as a public health measure to reduce the onset and progression of the disease. The present findings highlight the underlying pathophysiological association between vitamin C and AD. Clinical studies are warranted to elucidate its exact mechanistic role in AD pathophysiology and prevention.

## Data availability statement

The original contributions presented in this study are included in the article/Supplementary material, further inquiries can be directed to the corresponding author.

## Author contributions

SZ was responsible for the conceptualization of the study, data review and analysis, and manuscript preparation and editing. MH and SM were responsible for data collection (literature review), data entry, statistical analysis, data interpretation, and manuscript drafting and editing. SA contributed to the content of the article and manuscript editing and write-up. All authors contributed to the article and approved the submitted version.

## Abbreviations

CNS: Central nervous system
BDNF: brain-derived neurotrophic factor
CSF: cerebrospinal fluid
AD: Alzheimer’s disease
D β H: dopamine β-hydroxylase
A β: amyloid-β
APP: amyloid precursor protein
AA: ascorbic acid
AGEs: advanced glycation end products
RAGE: receptors for AGE
BBB: blood brain barrier
LRP-1: low-density lipoprotein receptor-1
GSH: glutathione
SSRIs: selective serotonin reuptake inhibitors
4-HNE: 4-hydroxynonenal
MCI: mild cognitive impairment
ROS: reactive oxygen species
CDR: Clinical Dementia Rating
HPA: hypothalamic–pituitary–adrenal
MMSE: Mini-Mental State Examination
CI: confidence interval
IQR: inter-quartile range
SMD: standard mean deviation.
